# Supplementary material for: vcfgl: a flexible genotype likelihood simulator for VCF/BCF files
Source: Bioinformatics. 2025 Mar 5;41(4):btaf098. doi: 10.1093/bioinformatics/btaf098 (PMC11968323; doi:10.1093/bioinformatics/btaf098)
Supplement: btaf098_Supplementary_Data [file btaf098_supplementary_data.zip › vcfgl_Manuscript.pdf]

*Bioinformatics*
doi.10.1093/bioinformatics/xxxxxx
Advance Access Publication Date: Day Month Year
Manuscript Category

OXFORD

Sequence analysis

# vcfgl: A flexible genotype likelihood simulator for VCF/BCF files

**Isin Altinkaya [1,*], Rasmus Nielsen [1,2*] and Thorfinn Sand Korneliussen [1,*]**

[1] Lundbeck Foundation GeoGenetics Centre, Globe Institute, University of Copenhagen, Copenhagen, Denmark. . [2] Departments of Integrative Biology and Statistics, University of California, Berkeley.

* To whom correspondence should be addressed.

Associate Editor: XXXXXXX

Received on XXXXX; revised on XXXXX; accepted on XXXXX

## Abstract

**Motivation:** Accurate quantification of genotype uncertainty is pivotal in ensuring the reliability of genetic inferences drawn from NGS data. Genotype uncertainty is typically modeled using Genotype Likelihoods (GLs), which can help propagate measures of statistical uncertainty in base calls to downstream analyses. However, the effects of errors and biases in the estimation of GLs, introduced by biases in the original base call quality scores or the discretization of quality scores, as well as the choice of the GL model, remain under-explored.
**Results:** We present vcfgl, a versatile tool for simulating genotype likelihoods associated with simulated read data. It offers a framework for researchers to simulate and investigate the uncertainties and biases associated with the quantification of uncertainty, thereby facilitating a deeper understanding of their impacts on downstream analytical methods. Through simulations, we demonstrate the utility of vcfgl in benchmarking GL-based methods. The program can calculate GLs using various widely used genotype likelihood models and can simulate the errors in quality scores using a Beta distribution. It is compatible with modern simulators such as msprime and SLiM, and can output data in pileup, VCF/BCF and gVCF file formats, supporting a wide range of applications. The vcfgl program is freely available as an efficient and user-friendly software written in C/C++.
**Availability:** vcfgl is freely available at `https://github.com/isinaltinkaya/vcfgl`.
**Contact:** isin.altinkaya@sund.ku.dk
**Supplementary information:** Supplementary data are available at *Bioinformatics* online.

## 1 Introduction

Next-generation sequencing (NGS) has enabled a deeper understanding of genetic variation across time and various biological systems, particularly non-model organisms and ancient populations (da Fonseca *et al.*, 2016). Accurately quantifying genotype uncertainty through genotype likelihoods is fundamental in ensuring the reliability of genetic inferences drawn from NGS data. This is particularly important in low-depth and ancient DNA data analysis, where the biases and genotype uncertainties are pronounced, and the probability of only sampling nucleotides from one chromosome in a diploid is non-negligible.

Genotype likelihoods provide a probabilistic measure of genotype uncertainty that can incorporate information on alignment or assembly uncertainty and base-calling uncertainty (Nielsen *et al.*, 2011). Genotype likelihoods are integral not only to calling genotypes but also to a multitude of downstream methods used in a diverse set of scientific inquiries, such as estimating relatedness (Korneliussen and Moltke, 2015; Waples *et al.*, 2019), allele frequency spectra (Korneliussen *et al.*, 2014; Mas-Sandoval *et al.*, 2022; Rasmussen *et al.*, 2022), calculating genetic distances (Vieira *et al.*, 2016; Zhao *et al.*, 2022), conducting Principal Component Analysis (Meisner and Albrechtsen, 2018), evaluating linkage disequilibrium (Fox *et al.*, 2019), and admixture proportions (Skotte *et al.*, 2013; Lou *et al.*, 2021; O’Rawe *et al.*, 2015).

Quantifying uncertainty using quality scores and genotype likelihoods is itself subject to uncertainties and potential biases. These can arise from errors and biases in estimating per-base error probabilities, the discretization of genotype likelihoods, or the choice of genotype likelihood model. Such factors introduce additional layers of bias and uncertainty, which may not have been considered or sufficiently addressed by existing

© The Author 2022. Published by Oxford University Press. All rights reserved. For permissions, please e-mail: journals.permissions@oup.com 

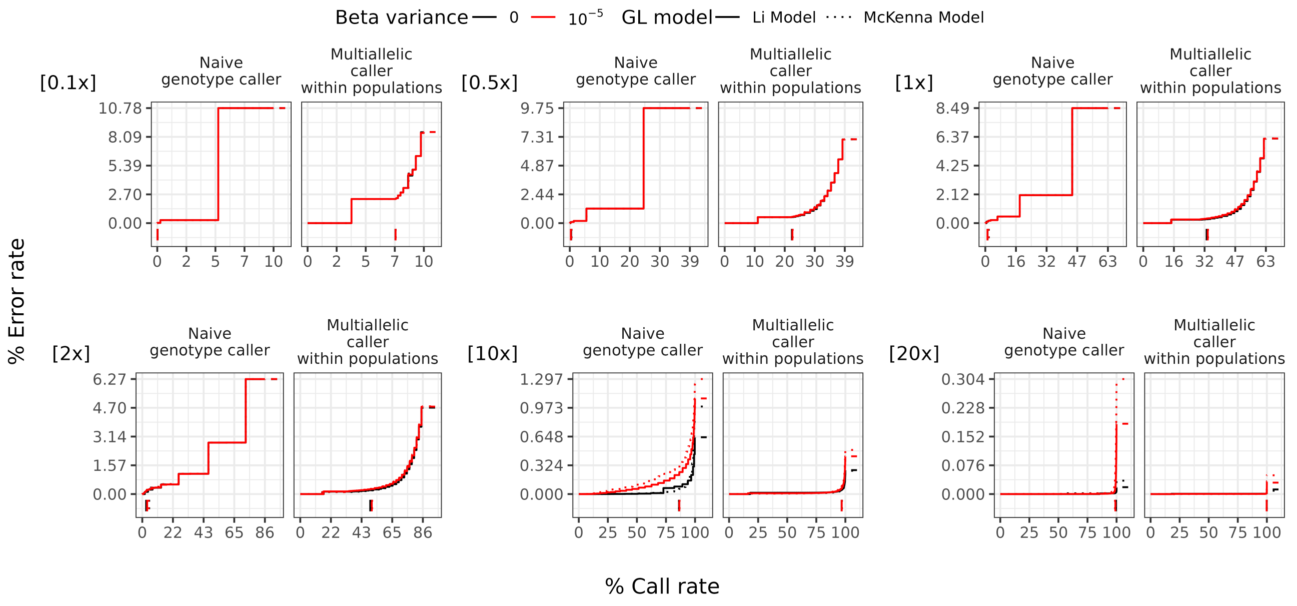


**Fig. 1.** Performance of genotype calling using the naive genotype caller method and BCFtools multiallelic caller with beta distributed errors in the estimation of the quality scores, by read depth. Colors indicate different variances in the beta distribution (0, and $10^{-5}$, respectively). The line types indicate the Li GL model (1), and McKenna GL model (2) (for details see the Supplementary Material, Section 1.4). The genotype calling error rates (y-axis) and call rates (x-axis) are defined in the main text. The average per-site read depth is indicated in the top left corner of each plot. The curves are obtained by varying the `GQ` threshold for genotype calling. The vertical line segments below 0 on the y-axis denote the minimum `GQ` threshold of 20, and the horizontal line segments after the final call rate on the x-axis denote the final error rate of each group. The data is from 20 replicates of 100 diploid individuals simulated using msprime, resulting in 328, 230 variable sites per simulation replicate (for details, see the Supplementary Material, Section 2). The BCFtools multiallelic caller is used for each population separately, and the prior parameter is disabled.

methods. Moreover, new sequencing platforms and continuous changes in sequencing technologies necessitate flexible tools for quantifying the effect of estimation uncertainty through simulations.

Existing tools for simulating genotype likelihoods, such as msToGlf (utility program in ANGSD package), have contributed to developing and evaluating various genotype likelihood-based methods (Zhao *et al.*, 2023; Soraggi *et al.*, 2018; Luqman *et al.*, 2021; Wang *et al.*, 2016; Mas-Sandoval *et al.*, 2022; Fox *et al.*, 2019; Korneliussen *et al.*, 2014). However, they lack the functionality for modeling uncertainty in the GL estimation that is central to vcfgl's function, such as realistic error modeling with Beta-distributed errors and platform-specific quality score binning. Moreover, they are not compatible with modern simulation tools and widely used file formats like VCF/BCF. This gap in the simulation capabilities limits researchers' ability to simulate complex data scenarios, benchmark methods, and examine the effects of the quantification of uncertainty in NGS data. These limitations underscore the need for modern and flexible tools to model these uncertainties and simulate data with complex scenarios.

## 2 Method

We introduce vcfgl, a lightweight utility tool for simulating genotype likelihoods. The program incorporates a comprehensive framework for simulating uncertainties and biases, including those specific to modern sequencing platforms. It offers compatibility with modern simulators such as msprime (Baumdicker *et al.*, 2022) and SLiM (Messer, 2013; Haller and Messer, 2023) through the use of VCF files. It is a lightweight tool that does not require many dependencies and is implemented in C/C++ for facilitating fast and efficient simulations. To our knowledge, vcfgl is the only tool for simulating genotype likelihoods offering this functionality. The resulting VCF files can then be used with many tools and frameworks, such as BCFtools (Danecek *et al.*, 2021), GATK (McKenna *et al.*, 2010; Van Der Auwera and O'Connor, 2020), and ANGSD (Korneliussen *et al.*, 2014).

Given a VCF file with genotypes, vcfgl can simulate sequencing data, quality scores, calculate the genotype likelihoods and various VCF tags, such as I16 and QS tags used in downstream analyses for quantifying the base calling and genotype uncertainty. For simulating sequence depth, vcfgl uses a Poisson distribution with a fixed mean. For simulating errors in the base-calling error probabilities, it utilizes a Beta distribution, which is routinely used in statistical models for modeling the variability of success in trials, making it suitable for modeling errors in base-calling error probabilities. The shape parameters are adjusted to obtain a distribution with a mean equal to the specified error probability and variance equal to a specified variance parameter (for more details, see Supplementary Material, Section 2). The program provides options for two commonly-used genotype likelihood models, the McKenna genotype likelihood model with independent errors (McKenna *et al.*, 2010), and the Li genotype likelihood model that models non-independent error structure and is used in SAMtools/BCFtools (Li *et al.*, 2008; Li, 2011). Detailed descriptions of the models can be found in Supplementary Material, Section 1.

The identification of the variable sites is itself subject to uncertainties, especially in the context of non-model organisms, low-depth sequencing, and ancient DNA data (Nielsen *et al.*, 2011). Furthermore, correct handling of invariant and missing sites in downstream analyses is important for the reliability of the conclusions drawn from genomic data. Consequently, the modern and widely utilized Variant Call Format (VCF), originally developed for storing variant information, has evolved to retain the information from invariable sites, thereby facilitating a comprehensive genomic overview. To address this, vcfgl provides the option to simulate the invariable sites, which is usually not possible to obtain directly from simulators. However, as the inclusion of these sites also presents a computational challenge due to the massive increase in the data volume,

modern file formats such as genomic VCF (gVCF) have been introduced to address this. The gVCF format uses a genomic block compression approach that can efficiently store invariant sites by grouping them into non-variant block records, thereby reducing the file size footprints (Caetano-Anolles, 2023). Our program can simulate invariable sites, and can output both VCF and gVCF files that are compatible with GATK and BCFtools gVCF formats, thereby allowing the user to both perform analyses incorporating invariable sites and test the effects of various SNP calling methods on downstream analyses.

## 3 Results and discussion

To demonstrate the utility of vcfgl, we benchmarked the accuracy of BCFtools multiallelic genotype calling method under different scenarios mimicking the classic Out-of-Africa model. We simulated variable sites in chromosome 22 for 100 diploid individuals using msprime (Baumdicker *et al.*, 2022) with 20 replicates (for more details, see Supplementary Material Section 2). We then used vcfgl to simulate genotype likelihoods and quality scores at read depths of 0.1, 0.5, 1, 2, 10, 20, and 100. We simulated the errors in quality scores using a mean error rate of 0.2% and beta distribution with variance parameters of 0 (no variance, i.e. precise quality scores) and $10^{-5}$. We calculated the associated genotype likelihoods using both Li (`-GL 1`) and McKenna (`-GL 2`) error models. We performed genotype and SNP calling using both naive genotype calling approach, and the BCFtools multiallelic caller (Danecek *et al.*, 2021). With the naive genotype caller, we pick the genotype corresponding to the highest genotype likelihood. We used the BCFtools multiallelic genotype caller with the '-P 0' option. The main difference between the two genotype calling methods is then that the BCFtools multiallelic caller identifies alleles (and thereby SNPs) prior to genotype calling and uses the allele frequencies, estimated using the read quality scores, in a Hardy-Weinberg equilibrium prior for both SNP and genotype calling (`https://samtools.github.io/bcftools/call-m.pdf`).

To evaluate the performance of genotype calling methods, we calculated two metrics for each simulation replicate for each individual: error rate and call rate. Here, call rate is defined as the proportion of sites with genotype calls out of the total number of sites simulated, based on a given threshold for `GQ`. The error rate is calculated as the count of wrongly called genotypes, standardized by the count of all genotype calls meeting the same call criteria. Detailed descriptions regarding the error rate and call rate calculation can be found in the Supplementary Material, Section 3.3.

Comparing the two genotype calling approaches, we observe that the additional step of identifying alleles (which includes SNP calling) in the BCFtools multiallelic genotype calling method results in more accurate genotype calls compared to the naive maximum likelihood approach that does not include a SNP calling step. We also observe that with the genotype likelihoods calculated using both McKenna and Li error models, as expected, the error rate decreases with increasing read depth (see Figure 1). The area under the curve (AUC) values calculated for each curve in Figure 1 reveal the overall relative performance differences, where we see that the differences between the two GL models become more pronounced as the read depth increases (see Figure S11 and Figure S12).

Using BCFtools multiallelic caller, we also tested two genotype calling approaches: genotype calling across populations, where the genotype calling is performed on the whole dataset, and within populations genotype calling, where the genotypes are called separately for each population, allowing for population-specific allele frequency estimation and the detection of population-specific variants. Across the different read depths, we observe that the within population approach performs better than the across populations approach (see Figure S1).

The maximum runtime for these simulations was 7 minutes 40 seconds per replicate without multi-threading, with a mean read depth of 20, when simulating errors in quality scores. Without simulating the quality score errors, the maximum run time was 1 minute 39 seconds. The simulation in both cases consisted of $328,230$ sites and 100 simulated diploid individuals. We addressed the bottleneck in the file writing step by using the HTSlib library's threading functionality to allow threading in the compression stream. Comparing the use of one thread versus four, we've observed a 13% reduction in processing time, down from 7 minutes 40 seconds to 6 minutes 47 seconds. The runtime and file size depend on the number of samples and the amount of sequence data simulated and, of course, on the disk IO. All analyses were conducted on a Red Hat Enterprise Linux 8.8 (Ootpa) system with an Intel(R) Xeon(R) Gold 6152 CPU at 2.10 GHz (x86_64), 754 GiB RAM, and a Linux 4.18.0-477.27.1.el8_8.x86_64 kernel. The benchmarking pipeline was implemented as a Snakemake workflow for reproducibility (Mölder *et al.*, 2021) and is freely available at `github.com/isinaltinkaya/vcfgl_benchmarking/`.

In our benchmarking analysis using real sequencing data from the 1000 Genomes Project, genotype discordance rates decreased with increasing sequencing depth (Figure S10). Simulations with vcfgl using realistic error rates yielded lower discordance rates than subsampled real data, highlighting the impact of sequencing errors and alignment artifacts in real datasets. The naive genotype calling method used here contrasts with the 1000 Genomes ground-truth genotypes, which incorporated population allele frequencies, contributing to the observed discrepancies. Additionally, the uneven depth distribution in real data contrasts with vcfgl's Poisson-simulated depth, which may partially explain the higher discordance in real data. These findings highlight the utility of vcfgl for benchmarking genotype calling methods while emphasizing the challenges posed by sequencing errors and depth variability in real datasets.

To our knowledge, msToGlf (a utility program within the ANGSD package (Korneliussen *et al.*, 2014)) is the only tool currently available for simulating genotype likelihoods. However, unlike vcfgl, msToGlf lacks the ability to simulate genotype likelihoods with realistic error probabilities, including Beta-distributed errors and base-calling quality score binning specific to various sequencing platforms. Additionally, vcfgl accepts VCF files with accurate genotypes as input, which can include observed genotype calls from real data or tree sequences simulated by popular tools such as msprime (Baumdicker *et al.*, 2022) and SLiM (Messer, 2013; Haller and Messer, 2023). In contrast, msToGlf relies solely on input from the ms program and simply calculates genotype likelihoods based on this input. Furthermore, msToGlf lacks compatibility with modern simulation tools and widely used file formats like VCF/BCF, which limits its applicability in simulating complex data scenarios, benchmarking methods, and examining the effects of uncertainty quantification in NGS data. These limitations underscore the unique capabilities of vcfgl, which offers advanced modeling of uncertainty, flexibility in input formats, and the ability to simulate realistic scenarios. As msToGlf does not incorporate these functionalities, a direct quantitative comparison with vcfgl would not provide meaningful insights into the utility or performance of vcfgl.

In addition to the models evaluated in this study, other genotype likelihood formulations could be relevant for future work, including the Atlas GL model, which incorporates post-mortem damage for ancient DNA data (Link *et al.*, 2017); the Maruki and Lynch GL model, which provides an alternative likelihood formulation to be used for genotype calling with low-coverage data (Maruki and Lynch, 2017); the Günther and Schraiber GL model, which applies empirical adjustments to GLs to mitigate mapping bias (Günther and Schraiber, 2024); and the SNPtools GL model, which uses a BAM-specific binomial mixture modeling approach for estimating GLs to handle data from heterogeneous platforms, reference bias, and low-quality data (Wang *et al.*, 2013). Future improvements may include modeling alignment and assembly-related

biases, for example using mappability maps, quantifying mapping biases, site-specific errors, and modeling correlated depth distributions, including localized low- and high-depth regions.

Our simulation tool, vcfgl, provides a framework for developing more accurate and reliable genetic data analysis methods, ultimately enhancing our understanding of genetic variations and their implications.

## Funding

This work was supported by Lundbeck Foundation Centre for Disease Evolution: [R302-2018-2155 to IA]; Centre for Ancient Environmental Genomics: [DNRF174 to TSK]; and; Carlsberg Foundation Young Researcher Fellowship awarded by the Carlsberg Foundation in 2019 [CF19-0712 to TSK].

*Conflict of interest:* None declared

## References

Baumdicker, F. *et al.* (2022). Efficient ancestry and mutation simulation with msprime 1.0. *Genetics*, **220**(3).

Caetano-Anolles, D. (2023). *GVCF - Genomic Variant Call Format Technical Documentation*.

da Fonseca, R. R. *et al.* (2016). Next-generation biology: Sequencing and data analysis approaches for non-model organisms. *Mar. Genomics*, **30**, 3–13.

Danecek, P. *et al.* (2021). Twelve years of SAMtools and BCFtools. *Gigascience*, **10**(2).

Fox, E. A. *et al.* (2019). ngsLD: evaluating linkage disequilibrium using genotype likelihoods. *Bioinformatics*, **35**(19), 3855–3856.

Günther, T. and Schraiber, J. G. (2024). Estimating allele frequencies, ancestry proportions and genotype likelihoods in the presence of mapping bias. *bioRxiv*, page 2024.07.01.601500.

Haller, B. C. and Messer, P. W. (2023). SLiM 4: Multispecies eco-evolutionary modeling. *Am. Nat.*, **201**(5), E127–E139.

Korneliussen, T. S. and Moltke, I. (2015). NgsRelate: a software tool for estimating pairwise relatedness from next-generation sequencing data. *Bioinformatics*, **31**(24), 4009–4011.

Korneliussen, T. S. *et al.* (2014). ANGSD: Analysis of next generation sequencing data. *BMC Bioinformatics*, **15**(1), 356.

Li, H. (2011). A statistical framework for SNP calling, mutation discovery, association mapping and population genetical parameter estimation from sequencing data. *Bioinformatics*, **27**(21), 2987–2993.

Li, H. *et al.* (2008). Mapping short DNA sequencing reads and calling variants using mapping quality scores. *Genome Res.*, **18**(11), 1851–1858.

Link, V. *et al.* (2017). ATLAS: Analysis tools for low-depth and ancient samples. *bioRxiv*, page 105346.

Lou, R. N. *et al.* (2021). A beginner's guide to low-coverage whole genome sequencing for population genomics. *Mol. Ecol.*, **30**(23), 5966–5993.

Luqman, H. *et al.* (2021). Identifying loci under selection via explicit demographic models. *Mol. Ecol. Resour.*, **21**(8), 2719–2737.

Maruki, T. and Lynch, M. (2017). Genotype calling from population-genomic sequencing data. *G3 (Bethesda)*, **7**(5), 1393–1404.

Mas-Sandoval, A. *et al.* (2022). Fast and accurate estimation of multidimensional site frequency spectra from low-coverage high-throughput sequencing data. *Gigascience*, **11**.

McKenna, A. *et al.* (2010). The genome analysis toolkit: a MapReduce framework for analyzing next-generation DNA sequencing data. *Genome Res.*, **20**(9), 1297–1303.

Meisner, J. and Albrechtsen, A. (2018). Inferring population structure and admixture proportions in low-depth NGS data. *Genetics*, **210**(2), 719–731.

Messer, P. W. (2013). SLiM: simulating evolution with selection and linkage. *Genetics*, **194**(4), 1037–1039.

Mölder, F. *et al.* (2021). Sustainable data analysis with snakemake. *F1000Res.*, **10**(33), 33.

Nielsen, R. *et al.* (2011). Genotype and SNP calling from next-generation sequencing data. *Nat. Rev. Genet.*, **12**(6), 443–451.

O'Rawe, J. A. *et al.* (2015). Accounting for uncertainty in DNA sequencing data. *Trends Genet.*, **31**(2), 61–66.

Rasmussen, M. S. *et al.* (2022). Estimation of site frequency spectra from low-coverage sequencing data using stochastic EM reduces overfitting, runtime, and memory usage. *bioRxiv*.

Skotte, L. *et al.* (2013). Estimating individual admixture proportions from next generation sequencing data. *Genetics*, **195**(3), 693–702.

Soraggi, S. *et al.* (2018). Powerful inference with the D-statistic on low-coverage whole-genome data. *G3 (Bethesda)*, **8**(2), 551–566.

Van Der Auwera, G. A. and O'Connor, B. D. (2020). *Genomics in the cloud: Using docker, gatk, and wdl in Terra*. O'Reilly Media.

Vieira, F. G. *et al.* (2016). Improving the estimation of genetic distances from next-generation sequencing data: Genetic distances from NGS data. *Biol. J. Linn. Soc. Lond.*, **117**(1), 139–149.

Wang, J. *et al.* (2016). Variation in linked selection and recombination drive genomic divergence during allopatric speciation of european and american aspens. *Mol. Biol. Evol.*, **33**(7), 1754–1767.

Wang, Y. *et al.* (2013). An integrative variant analysis pipeline for accurate genotype/haplotype inference in population NGS data. *Genome Res.*, **23**(5), 833–842.

Waples, R. K. *et al.* (2019). Allele frequency-free inference of close familial relationships from genotypes or low-depth sequencing data. *Mol. Ecol.*, **28**(1), 35–48.

Zhao, D. *et al.* (2023). A genomic quantitative study on the contribution of the ancestral-state bases relative to derived bases in the divergence and local adaptation of populus davidiana. *Genes (Basel)*, **14**(4).

Zhao, L. *et al.* (2022). DistAngsd: Fast and accurate inference of genetic distances for next-generation sequencing data. *Mol. Biol. Evol.*, **39**(6).
